# Risk factors for diabetic kidney disease in adults with longstanding type 1 diabetes: results from the Canadian Study of Longevity in Diabetes

**DOI:** 10.1080/0886022X.2019.1614057

**Published:** 2019-06-04

**Authors:** Nigar Sekercioglu, Leif Erik Lovblom, Petter Bjornstad, Julie A. Lovshin, Yuliya Lytvyn, Geneviève Boulet, Mohammed A. Farooqi, Andrej Orszag, Vesta Lai, Josephine Tse, Leslie Cham, Hillary A. Keenan, Michael H. Brent, Narinder Paul, Vera Bril, Bruce A. Perkins, David Z. I. Cherney

**Affiliations:** a Department of Medicine, Division of Nephrology, University of Toronto, Toronto, Canada;; b Department of Health Research Methods, Evidence, and Impact, McMaster University, Hamilton, Canada;; c Lunenfeld-Tanenbaum Research Institute, Mount Sinai Hospital, Toronto, Canada;; d Department of Pediatrics, Division of Endocrinology and Department of Medicine, Division of Nephrology, University of Colorado School of Medicine, Aurora, CO, USA;; e Department of Medicine, Division of Endocrinology and Metabolism, Sunnybrook Health Sciences Centre, University of Toronto, Toronto, Canada;; f Research Division, Joslin Diabetes Center, Boston, MA, USA;; g Department of Ophthalmology and Vision Sciences Faculty of Medicine, University of Toronto, Toronto, Canada;; h Joint Department of Medical Imaging, University of Toronto, Toronto, Canada and Department of Medical Imaging, Western University, London, Canada;; i Department of Medicine, Division of Neurology, University Health Network, University of Toronto, Toronto, Canada;; j Department of Medicine, Division of Endocrinology and Metabolism, Mount Sinai Hospital, University of Toronto, Toronto, Canada;; k Department of Physiology, University of Toronto, Toronto, Canada;; l Department of Physiology and Banting and Best Diabetes Centre, University of Toronto, Toronto, Canada

**Keywords:** Type 1 diabetes, diabetic kidney disease, diabetic neuropathy, diabetic retinopathy

## Abstract

**Objectives:** Diabetic kidney disease (DKD) is an independent predictor of cardiovascular morbidity and mortality in type 1 diabetes (T1D). We aimed to explore clinical and biochemical factors, including the achievement of American Diabetes Association (ADA) recommended targets associated with DKD in people living with T1D for ≥50 years.

**Methods:** This was a post hoc analysis of a cross-sectional study of 75 participants enrolled in the Canadian Study of Longevity in T1D. We explored diabetes-related complications, including neuropathy, retinopathy, cardiovascular disease, and DKD. Study participants were dichotomized based on the achievement of ADA recommended targets as the low-target group (achieving ≤4 targets, *n* = 31) and high-target group (achieving >4 targets, *n* = 44). The outcome of interest was DKD defined by estimated glomerular filtration rate (eGFR) values <60/mL/min/1.73 m^2^ and/or 24-h albumin excretion >30 mg. Multivariable logistic regression models were employed to estimate odds ratios (ORs) for DKD with 95% confidence intervals (CIs).

**Results:** Of the 75 participants with prolonged T1D duration (45% male, mean age 66 years), 25 participants had DKD and 50 did not. There was no statistical difference between the high- and low-target groups in terms of age and body mass index. eGFR was significantly higher and the prevalence of diabetic retinopathy was significantly lower in the high-target group. Older age at diagnosis of T1D and lower frequency component to high-frequency component ratio increased the odds of having DKD.

**Conclusions:** In adults with prolonged T1D duration, older age at diagnosis and lower heart rate variability may be associated with DKD.

## Introduction

Despite modern advances in replacing (human insulin analogues) and delivering insulin (continuous subcutaneous insulin infusion, hybrid-loop systems, artificial pancreas) and monitoring glycemic control (self-monitoring of blood glucose, continuous glucose monitoring), mortality remains high in people with type 1 diabetes (T1D) [1–3]. Furthermore, microvascular and macrovascular complications are increasingly common after prolonged T1D disease duration, though some people, for reasons that are not clear, are resistant to the development of such chronic complications [4–6].

Optimal metabolic control is critical for avoiding short and long-term complications in the setting of T1D, including maintenance of normoglycemia with basal and prandial insulin management. For example, in the Diabetes Control and Complications Trial, intensive glycemic control was associated with a reduction in the risk of developing diabetic kidney disease (DKD), which is detected through routine monitoring of albumin excretion and estimated glomerular filtration rate (eGFR) annually [7–9]. Observational studies also support the link between glycemic control and microvascular complications, including DKD in T1D [10–13]. Unfortunately, DKD remains common in the setting of T1D, and increases the risk of cardiovascular disease (CVD) and end stage kidney disease [14–16]. As a consequence, approximately 30% of patients with T1D exhibit clinical evidence of DKD [17–19], reflected by low glomerular filtration rate or proteinuria [20].

Overall, both non-modifiable (i.e., age, sex, age of onset and genetic predisposition) and modifiable risk factors (e.g., optimization of renin–angiotensin aldosterone system (RAAS) inhibition and subsequent achievement of glycemic and blood pressure targets) contribute to the initiation and progression of kidney complications in diabetes [21,22]. We aimed to explore which clinical and biochemical factors, including the achievement of ADA recommended targets associated with DKD in people living with T1D for ≥50 years. We also examined the relationship between the achievement of ADA targets and diabetic complications.

## Methods

### Study population and data collection

This was a cross-sectional study of 75 people enrolled in the second phase of the Canadian Study of Longevity in T1D. Participants with T1D were recruited among the 300 participants who took part in the mail-based survey based on geographical proximity to Toronto General Hospital and willingness to participate [23–25].

Inclusion criteria for the participants included: (1) duration of ≥50 years of T1D and (2) ability to understand and cooperate with study procedures. Those with any current eye infection, corneal damage, severe movement disorder, or proparacaine allergy that would preclude safe *in vivo* corneal confocal microscopy examination were excluded. The study participants were recruited consecutively. All participants provided written informed consent, and the study and its procedures were approved by the institutional ethics boards at the University Health Network and Mount Sinai Hospital in Toronto.

Patient’s demographics (age, sex, age at diagnosis) and clinical characteristics were extracted from the Longevity dataset. Clinical characteristics included body mass index (BMI), blood pressure parameters (systolic and diastolic blood pressure (SBP and DBP, respectively)) and laboratory values (hemoglobin A1C (A1C), low-density lipoprotein (LDL), high-density lipoprotein (HDL), triglyceride levels, albumin to creatinine ratio (ACR)). Blood pressure was measured in duplicate with an automated sphygmomanometer (53000 Vital Signs Monitor; Welch-Allyn Inc., Skaneateles Falls, NY) while blood was drawn for analysis. The average of two values was used to assess SBP and DBP at each time point.

### Definition of complications

The diabetes-related chronic complications evaluated in the Longevity study included neuropathy, retinopathy, macrovascular disease, and DKD. The presence of diabetic peripheral polyneuropathy was established using consensus criteria which consisted of abnormal nerve conduction studies (nerve conduction velocity (NCV) and amplitude potentials) in the sural and peroneal nerves (representing sensory and motor innervation, respectively), corroborated by the presence of clinical signs and/or symptoms using the Toronto Clinical Neuropathy Score (TCNS) grading system [26,27]. The TCNS grading system is validated for peripheral sensorimotor polyneuropathy and examines superficial and deep sensation [26]. The severity of neuropathy is graded based on the total score from the scale as mild (TCNS 5–8), moderate (TCNS 9–11), or severe (TCNS ≥12) [26].

We measured NCVs and amplitude potentials (NCA) for the sural sensory and peroneal motor nerves using a needle electromyography. Subsequently, we recorded following nerve function parameters: sural nerve amplitude potential (SNAP), sural nerve conduction velocities (SNCVs), peroneal nerve amplitude potential (PNAP), and peroneal nerve conduction velocities (PNCVs).

We examined cardiac autonomic function with frequency-domain and time-domain analyses of heart rate variability (HRV) which is measured by the variation in consecutive heart beats from two short-term values (two 10-min measurements of the electrocardiography). High variation implies high parasympathetic tone and adequate adaptation to micro-environmental changes while low variation implies low parasympathetic tone. First, we calculated time domain parameters using the root mean square of successive heartbeat interval differences (RMSSDs, parasympathetic) and the standard deviation of normal-to-normal intervals (SDNN, sympathetic). The spectral component of HRV analysis employs frequency-domain parameters – the lower frequency component (LF, sympathetic and parasympathetic) and high-frequency component (HF, parasympathetic) – and is a measure of sympathovagal tone [28]. LF and HF were measured as relative power of the low- and high frequency bands in normal units [29]. The methods used for these measurements were previously described elsewhere [30].

Retinal imaging was performed at the Toronto Western Hospital in the Donald K. Johnson Eye Centre. The presence of retinopathy was defined on the basis of imaging results for the presence of proliferative or non-proliferative morphological changes and Early Treatment of Diabetic Retinopathy Study classification. Severity of retinopathy was graded by retinal photographs using Optos 200Tx Ultra-widefield digital retinal imaging (Optos, Dunfermline, UK) and measurement of macular thickness and presence or absence of diabetic macular edema was performed using optical coherence tomography (CIRRUS HD-OCT; Zeiss, Oberkochen, Germany).

Cardiovascular disease was defined from self-report including history of angina, myocardial infarction, cardiac bypass, or angioplasty surgery. Peripheral vascular disease (PVD) was ascertained separately from CVD and was defined as a history of bypass/angioplasty for peripheral arteries.

### ADA recommended targets

Risk-factor scores based on American Diabetes Association (ADA) recommended targets were established as follows: A1c <7%, SBP <140 mmHg, DBP <90 mmHg, LDL ≤1.80 mmol/L (70 mg/L), HDL >1 mmol/L in males (40 mg/dL) and >1.3 mmol/L (50 mg/dL) in females, and triglyceride ≤1.7 mmol/L (150 mg/dL). Study participants were dichotomized according to the achievement of ADA recommended targets as the low-target group (achieving ≤4 targets, *n* = 28) and high-target group (achieving >4 targets, *n* = 47).

### Outcome variable

Participants with T1D were categorized as DKD resistor if they had eGFR_MDRD_ ≥60 mL/min/1.73 m^2^ and 24-h urine albumin excretion <30 mg/day at their screening visit; otherwise, they were assigned to the DKD group.

### Statistics and analysis plan

We used mean (standard deviation, SD) for normally distributed and median (inter-quartile range; IQR) for non-normally distributed data to describe the between-group differences. Furthermore, frequencies (numbers) and proportions were used for categorical variables to explore group differences. Logistic regression, including univariable, multivariable, and reduced models, was performed to test the hypothesis. We employed the Wald statistic for tests of significance of individual coefficients. Odds ratios (ORs) with 95% confidence intervals (CIs) were obtained. In the multivariable models, we included covariates that met a univariable criteria of alpha of 0.20. The reduced multivariable model used a backward variable selection approach. Two-sided tests were employed with a significance level of .05 in our models. Analyses were performed in SAS (version 9.4, SAS Institute, Cary, NC).

## Results

We included 75 patients with T1D (34 males, 41 females; 65.8 years ± 8 years) with prolonged duration (>50 years) in this cross-sectional study (Table 1). Among these, 25 patients had DKD and 50 did not. A total of 44 patients (58%) were included in the low-target group (10 males, 21 females; 66.5 years, ±8.2 years), while 31 patients (41%) in the high-target group (24 males, 20 females; 65.3 years, ±7.5 years). There were no differences in mean age (65 ± 7 vs. 67 ± 8 years, *p* = .30) between the groups. The proportion of males was significantly higher in the low-target group (55% vs. 32% men, *p* = .05). There was no significant difference in 24-h urine albumin excretion levels between the groups (36 ± 110 vs. 88 ± 230 mg/24 h, *p* = .18) while urine ACR at random urine samples was significantly lower (3.46 ± 8 vs. 9.5 ± 18, *p* = .05) and eGFR was significantly higher in the high-target group (76 ± 17 vs. 65 ± 15 mL/min/1.73 m^2^, *p* = .007) (Table 1).

**Table 1.** Characteristics of the study participants, according to achievement of guideline recommended targets.

Variables	All patients	High-target group >4 targets met (*n* = 44)	Low-target group ≤4 targets met (*n* = 31)	*p* Value
Patient’s demographics and clinical variables				
Age (years); mean (SD)	65.8 ± 8	65.3 ± 7.5	66.5 ± 8.2	.49
Male sex; *n* (%)	34 (45%)	24 (55%)	10 (32%)	.05
Duration of type 1 diabetes (years); median and interquartile range	54.6 [52,58]	54.0 [51.0,58.0]	54.5 [52.5,58.5]	.26
Age at diagnosis; median and interquartile range	10 [6,17]	10.0 [6.0,16.0]	10.5 [6.0,17.5]	.68
Weight (kg); mean (SD)	72.6 ± 12.1	71.72 ± 12.69	74.10 ± 11.16	.42
BMI; kg/m^2^; mean (SD)	26.6 ± 3.8	26.2 ± 4.0	27.4 ± 3.5	.14
SBP (mmHg); mean (SD)	131.5 ± 15.9	127 ± 10	137 ± 19	<.001
DBP (mmHg); mean (SD)	67.57 ± 5.02	67.27 ± 5.15	67.88 ± 4.85	.31
Heart rate (bpm); mean (SD)	66.2 ± 10.19	66.87 ± 10.54	65.16 ± 9.71	.41
Total daily insulin (units); mean (SD)	35.6 ± 13.2	36 ± 10.75	35 ± 15.96	.79
TCNS total score; median and interquartile range	6.0 [4.0,10.0]	7 (4,10)	5.5 (3.5,10.5)	.66
Biochemical characteristics				
A1C, *n* (%)	7.35 ± 0.83	7.13 ± 0.82	7.71 ± 0.74	.003
LDL (mmol/L); mean (SD)	1.86 ± 0.54	1.69 ± 0.5	2 ± 0.5	.001
HDL (mmol/L); mean (SD)	1.65 ± 0.45	1.68 ± 0.42	1.61 ± 0.49	.37
eGFR_MDRD_ (mL/min/ 1.73 m^2^); mean (SD)	72.25 ± 17.23	76 ± 17	65 ± 15	.007
ACR (spot); mean (SD)	5.94 ± 1.55	3.46 ± 8	9.5 ± 18	.05
Albumin excretion (mg/ 24 h); mean (SD)	58 ± 19	36 ± 110	88 ± 230	.18
Complications				
DKD present; *n* (%)	25 (33%)	12 (27%)	13 (40%)	.18
Neuropathy present; *n* (%)	65 (89%)	39 (88%)	26 (84%)	.89
Retinopathy present (PDR and non-PDR); *n* (%)	63 (84%)	34 (77%)	29 (93%)	.05
PDR present; *n* (%)	39 (52%)	19 (44%)	20 (64%)	.17
Cardiovascular disease present; *n* (%)	15 (20%)	7 (15%)	8 (25%)	.29
Nerve conduction studies and markers for heart rate variability				
SNCV (μV), mean (SD)	36 ± 6.2	37 ± 6.3	34 ± 5.8	.10
SNAP (μV), mean (SD)	2.93 ± 2.91	3.53 ± 3.22	2.03 ± 2	.03
PNAP (μV), mean (SD)	1.08 ± 1.49	1.97 ± 1.35	1.57 ± 1.66	.39
PNCV (μV), mean (SD)	36 ± 7.89	37 ± 7.2	34 ± 8.8	.14
SDNN (ms); mean (SD)	39 ± 34	38 ± 34	41 ± 34	.61
RMSSD (ms); mean (SD)	28.88 ± 28.42	25.7 ± 24.6	33.1 ± 32.7	.16
Low frequency normalized unit (nu); mean (SD)	346.09 ± 713.46	309 ± 729	400 ± 708	.59
High frequency normalized unit (nu); mean (SD)	282.31 ± 725.18	259 ± 786	315 ± 635	.75
Low frequency/high frequency ratio; mean (SD)	2.46 ± 2.12	2.22 ± 1.84	2.87 ± 2.62	.21

ACR: albumin to creatinine ratio; A1C: hemoglobin A1C; BMI: body mass index; DBP: diastolic blood pressure; DKD: diabetic kidney disease; DSPN: diabetic sensorimotor polyneuropathy; HF: high-frequency band; LF: low-frequency band; NPDR: non-proliferative diabetic retinopathy; PDR: proliferative diabetic retinopathy; PNAP: peroneal nerve action potential; PNCV: peroneal nerve conduction velocity; RMSDDs: root mean square of successive heartbeat interval differences; SD: standard deviation; SDNN: standard deviation of normal-to-normal intervals; SNAP: sural sensory nerve action potential; SNCV: sural sensory nerve conduction velocity; SBP: systolic blood pressure; TCNS: Toronto Clinical Neuropathy Score.

Significance at *p* < .05.

A total of 65 individuals met the criteria for neuropathy diagnosis, while 63 individuals had proliferative or non-proliferative diabetic retinopathy in this study cohort. Fifteen participants reported no prior diagnosis or knowledge of CVD. The prevalence of retinopathy was significantly lower in the high-target group. Nevertheless, other diabetes-related complications were not different between the groups (Table 1).

Group means for measures of neuropathy (PNAP, PNCV, and SNCV) did not significantly vary in the low and high target groups; however, SNAP was significantly higher in the high-target group (3.53 ± 3.22 vs. 2.03 ± 2 μV, *p* = .03). Time and frequency domain analyses of HRV did not differ between the groups (Table 1).

The results of the multivariable logistic regression analyses for variables that were associated with DKD are described in Table 2. In our study population, older age at diagnosis was associated with DKD (OR  = 1.08, 95% CI: 1.05–1.19). There was also a significant association between increased risk of having DKD and LF/HF ratio (OR  = 0.67, 95% CI: 0.47–0.95). As a result, lower LF/HF ratio was related to the likelihood of having DKD even after adjusting for age at diagnosis of T1D (Table 2).

**Table 2.** Results of the multivariable logistic regression, outcome DKD.

Variable	OR (95% CI)	*p* Value
Model 1 (with all variables from our initial model)		
Age	0.90 (0.78–1.04)	.17
Age at diagnosis	1.18 (1.02–1.37)	.02
PNAP	0.44 (0.19–1)	.06
SNAP	0.64 (0.40–1)	.06
SNCV	1.34 (1.08–1.65)	.007
LF/HF ratio	0.62 (0.39–0.99)	.04
Model 2 (backward selection approach)		
Age at diagnosis	1.08 (1.03–1.17)	.03
SNCV	1.03 (0.94–1.13)	.45
LF/HF ratio	0.64 (0.44–0.93)	.02
Model 3 (backward selection approach)		
Age at diagnosis	1.08 (1.05–1.19)	.04
LF/HF ratio	0.67 (0.47–0.95)	.02

CI: confidence interval; HF: high-frequency band; LF: low-frequency band; PNAP: peroneal nerve action potential; PNCV: peroneal nerve conduction velocity; OR: odds ratio; SNAP: sural sensory nerve action potential; SNCV: sural sensory nerve conduction velocity.

*Note.* We used a backward variable selection approach and included covariates that met a univariable criteria of alpha of 0.20.

Significance at *p* < .05.

## Discussion

In this analysis, we studied 75 adults with T1D, which we categorized into a high and low target groups. The differences in the groups included: (1) sex distribution was different between the groups with male dominance in the high-target group; (2) eGFR was higher in the high target group although the prevalence of DKD was not different between the groups; (3) the prevalence of diabetic retinopathy was lower in the high-target group; (4) there were significant differences between the low and high target groups in neuropathy, specifically for the larger nerve fiber function as evident in sural sensory nerve amplitude potentials; (5) older patients at diagnosis and patients with lower LF/HF ratio exhibited a greater odds of having DKD.

A recent large registry-based study showed an association between younger age at diagnosis and cardiovascular morbidity and mortality [31]. In line with these findings, two other studies reported excess mortality rates in early onset T1D [32,33]. There are several mechanisms that may explain the effect of age of onset on patient important outcomes in T1D [34]. First, age of onset is an intermediate outcome in the pathway of several exposure variables and healthy years expectancy [34]. Total glycemic burden and disease burden are expected to be higher in patients diagnosed at earlier ages. The rate and extent of beta cell destruction may also differ [31]. Consideration of age of onset may raise the possibility for delivering early cardiovascular or renal protective agents (e.g., renin–angiotensin–aldosterone system blockage and statins) in selected populations. On the other hand, patients with long-standing T1D in our study population may have different risk trajectories due to survival bias as our study subjects included patients that survived with T1D survived for more than 50 years, thereby limiting the generalizability of our results. Importantly, patients in this analysis had a long duration of disease, but a wide range of age at diagnosis making it difficult to compare and contrast with patients solely on the basis of having a young age at diagnosis.

The presence and severity of autonomic dysfunction have been linked with increased cardiovascular risk and also with alterations in heart rate blood pressure through changes in parasympathetic and sympathetic tone [35]. Heart rate variability is considered as an important feature of cardiac autonomic neuropathy in patients with diabetes. A recent systematic review showed a decrease in both parasympathetic and sympathetic activity with lower RMSSD, SDNN, LF, and HF in those with type 2 diabetes as compared to healthy controls [36]. However, in this study population, LF/HF ratios did not differ between the groups implying that among those adults with T1D, there are comparable loses in both components of the autonomic nervous system [36]. Although not statistically significant, SDNN, RMSSD, LF, HF, and LF/HF ratio were lower in the high target group as compared to the low target group in this study cohort. These results also suggest possible sympathetic dominance in those with poor metabolic control. Nevertheless, our logistic regression models showed lower LF/HF increases the likelihood for DKD.

There are several strengths of our study, including the gold-standard methods we used to characterize the prevalence and severity diabetes-related complications. We employed electrophysiological parameters of nerve conduction studies and clinical criteria to determine the presence and sub-type of peripheral diabetic neuropathy. To examine cardiac autonomic function, we measured time domain variables with indexes of total variability (SDNN) and beat-to-beat index (RMSSD) as well as frequency domain variables. The limitations of our study include small sample size and simultaneous measurement of exposure and outcome variables. Therefore, our results can be used only for hypothesis generation. We employed clinical and electrophysiological techniques to diagnose diabetic neuropathy, but did not explore structural changes using nerve biopsy, ultrasonography, or magnetic resonance imaging techniques. We also acknowledge that 24-h recordings likely provide more reliable assessment for HRV as compared short-term measurements.

Furthermore, this study’s dataset includes a cohort of T1D patients with long-survival. The diagnosis of PVD was confirmed based on previous history of bypass/angioplasty for peripheral arteries. However, we did not measure ankle-arm blood pressure indices for the screening and diagnose of PVD. Moreover, we did not explore carotid intima media thickness, an important marker for risk stratification for CVD [37]. Lastly, red cell distribution width has been associated with renal disease progression in those with DKD and we did not include this prognostic indicator in our study [38].

## Conclusions

After prolonged T1D duration, the prevalence diabetic retinopathy was lower in the high-target group. Older age at diagnosis with T1D and lower LF/HF can be associated with higher likelihood of DKD in long-standing T1D. These findings highlight the utility of risk stratification based on age at diagnosis in people with T1D. Large-scale longitudinal studies needed to explore the link between well-metabolic control and long-term diabetes complications.

## Author contributions

NS designed the study, researched, wrote, performed the statistical analyses, contributed to discussion, and reviewed/edited the manuscript; LEL contributed to the discussion, performed the statistical analyses, and reviewed/edited the manuscript; DZIC designed the study, researched, wrote, contributed to discussion, and reviewed/edited the manuscript; PB, JAL, YL, GB, MAF, AO, VL, JT, LC, HAK, MHB, NP, VB, and BAP contributed to discussion, and reviewed/edited the manuscript.
